# Identifying models of HIV care and treatment service delivery in Tanzania, Uganda, and Zambia using cluster analysis and Delphi survey

**DOI:** 10.1186/s12913-017-2772-4

**Published:** 2017-12-06

**Authors:** Sharon Tsui, Julie A. Denison, Caitlin E. Kennedy, Larry W. Chang, Olivier Koole, Kwasi Torpey, Eric Van Praag, Jason Farley, Nathan Ford, Leine Stuart, Fred Wabwire-Mangen

**Affiliations:** 1 Department of International Health, Johns Hopkins Bloomberg School of Public Health, 615 N. Wolfe Street, Baltimore, MD USA; 2 Department of Medicine – Infectious Diseases, Johns Hopkins University School of Medicine, 733 N. Broadway, Baltimore, MD USA; 3 Clinical Sciences Department, Institute of Tropical Medicine, Nationalestraat 155, 2000 Antwerp, Belgium; 4 Department of Clinical Research, London School of Hygiene and Tropical Medicine, Keppel St, London, UK; 5 School of Public Health, University of Ghana College of Health Sciences, Legon Boundary, Accra, Ghana; 6 Technical Support Division, Global Health Population and Nutrition, FHI 360, Karibu St., Haile Selassie Rd., Oysterbay, Dar es Salaam, Tanzania; 7 Department of Community – Public Health, Johns Hopkins University School of Nursing, 525 N. Wolfe St, Baltimore, MD USA; 8 University of KwaZulu Natal, King George V Ave, Durban, 4041 South Africa; 9 Association of Nurses in AIDS Care, 3538 Ridgewood Rd, Akron, OH USA; 10 Dept HIV, World Health Organization, Ave. Appia 20, 1211, 27 Genève, Switzerland; 11 FHI 360 (retired), 1825 Connecticut Ave NW, Washington, DC USA; 12 Department of Epidemiology and Biostatistics, Makerere University School of Public Health, New Mulago Hill Rd, Kampala, Uganda

**Keywords:** Africa, Antiretroviral therapy, Cluster analysis, Delphi method, Human resources for health, Task sharing, Task shifting

## Abstract

**Background:**

Organization of HIV care and treatment services, including clinic staffing and services, may shape clinical and financial outcomes, yet there has been little attempt to describe different models of HIV care in sub-Saharan Africa (SSA). Information about the relative benefits and drawbacks of different models could inform the scale-up of antiretroviral therapy (ART) and associated services in resource-limited settings (RLS), especially in light of expanded client populations with country adoption of WHO’s test and treat recommendation.

**Methods:**

We characterized task-shifting/task-sharing practices in 19 diverse ART clinics in Tanzania, Uganda, and Zambia and used cluster analysis to identify unique models of service provision. We ran descriptive statistics to explore how the clusters varied by environmental factors and programmatic characteristics. Finally, we employed the Delphi Method to make systematic use of expert opinions to ensure that the cluster variables were meaningful in the context of actual task-shifting of ART services in SSA.

**Results:**

The cluster analysis identified three task-shifting/task-sharing models. The main differences across models were the availability of medical doctors, the scope of clinical responsibility assigned to nurses, and the use of lay health care workers. Patterns of healthcare staffing in HIV service delivery were associated with different environmental factors (e.g., health facility levels, urban vs. rural settings) and programme characteristics (e.g., community ART distribution or integrated tuberculosis treatment on-site).

**Conclusions:**

Understanding the relative advantages and disadvantages of different models of care can help national programmes adapt to increased client load, select optimal adherence strategies within decentralized models of care, and identify differentiated models of care for clients to meet the growing needs of long-term ART patients who require more complicated treatment management.

**Electronic supplementary material:**

The online version of this article (10.1186/s12913-017-2772-4) contains supplementary material, which is available to authorized users.

## Background

Critical shortages and inefficient distributions of trained health workers constrains timely and universal access to HIV treatment in SSA [1]. Many antiretroviral therapy (ART) programmes have coped with staff shortages by extending the scope of practice for existing health workers [2–14], and creating new auxiliary cadres, including peer health workers for home-based patient monitoring [15–17], adherence supporters for clinic-based adherence counselling and home-based patient tracing [2, 15, 18–20], and expert patients and community health workers to relieve nurses of administrative tasks, such as patient file retrieval or archival, patient registration, and clinic navigation [21–23].

Redistributing tasks within health workforce involves task-shifting and task-sharing. **Task-shifting** is moving tasks from one cadre to another – usually from more to less specialized cadres (e.g., moving ART prescription from doctors to clinical officers (COs) or nurses) [24–26]. **Task-sharing** is a “team-based approach” where “medical care [is] provided to a patient by a set group (team) of different health professionals with different roles that maximize the skills and abilities of each team member” [27]. For example, COs and nurses may initiate ART and monitor stable patients while doctors manage patients with complex opportunistic infections.

Task-shifting and task-sharing in ART services vary tremendously across countries and programmes. To date, research has mainly focused on the safety and effectiveness of task-shifting [2, 4, 9, 11, 13, 16, 28–30]. Less is known about the relationship between clinic staffing patterns (e.g., who delivers services), the range of HIV care and treatment services offered, and the context in which task-shifting/task-sharing occur (e.g., types of health facilities). Understanding these relationships will identify different models of HIV care, which can be compared on their frequency of use, cost, and association with HIV-related treatment outcomes. Information about benefits and drawbacks of different HIV care models can also inform ART scale-up in RLS, especially following the World Health Organization’s (WHO) recommendation for immediate ART initiation of all HIV-positive individuals [31]. Country adoption of this recommendation should lead to increased client volumes and higher proportions of patients who start ART when healthier, underscoring the need to identify successful task-shifting/task-sharing strategies so countries can achieve the ultimate goal of providing lifelong ART and associated care for every HIV-infected person.

In this study, we sought to identify ART task-shifting/task-sharing service delivery models and describe their health facility and environmental characteristics. A cluster analysis of cross-sectional data from 19 diverse ART clinics in Tanzania, Uganda, and Zambia was conducted. Models of care were then verified by 18 experts using the Delphi survey process.

## Methods

### Study design and setting

Cross-sectional data for this analysis came from an ART retention and adherence study in Tanzania, Uganda, and Zambia led by FHI 360 (2008–2012). The main study’s purpose was to characterize retention and adherence rates in 19 ART clinics, and to examine programmatic and individual factors related to retention and adherence among adult patients on ART for at least 6 months. Clinics were purposefully selected with country partners and Ministries of Health (MOH) to include those with ≥300 patients from different socioeconomic and urban-rural locations with varying characteristics, including public/private/faith-based organizations, primary/secondary/tertiary-levels, and different ART adherence and provision strategies. Additional site selection details have been reported elsewhere [32, 33]. Information on the 19 ART clinics’ task-shifting/task-sharing characteristics was collected in a cross-sectional survey with ART clinic managers in 2010–2011 (Additional file 1).

### Measures

Trained interviewers used a structured survey to systematically document task-shifting/task-sharing practices at each clinic on the following ART-related services on a typical day: 1) registration, 2) initial patient triage, 3) initial ART prescription based on ART eligibility assessment, 4) ART monitoring and management (including drug side effects, CD4 cell viral load monitoring), 5) clinical services (diagnosis and management of opportunistic infections and management of common non-HIV conditions, i.e. malaria), 6) referral services, 7) ART dispensing, 8) counselling on ART adherence, 9) phlebotomy for laboratory testing, 10) follow-up of patients who missed appointments, and 11) medical records management. Data were stored in EpiData 3.1.

### Cluster analysis

We conducted a cluster analysis to identify models of ART task-shifting/task-sharing service delivery. All statistical analyses used SAS 9.3.

We first ran frequencies on all variables to assess variability, defined as differences observed in at least three of the 19 ART clinics. Only 33 of 69 initial variables had enough variability to be included in the analysis.

We then performed agglomerative hierarchical clustering using the Ward Method and the Russel and Rao Index as the measure of similarity – an algorithm selected for the asymmetric nominal nature of the data. The number of clusters were determined based on examination of the generated dendrogram and statistics measuring the cluster fit (pseudo F-statistic and pseudo T^2^ statistic). Nine sensitivity analyses were performed using other measures of similarity (e.g., Jaccard coefficient and Dice coefficient) and hierarchical methods (average, single, and complete) fitting for the asymmetric binary data, enhancing the reliability of the findings [34–36]. A cluster analysis based on the Ward method and the Russel and Rao Index was used to create the final solution as: 1) the Ward method compared to average, single, and complete hierarchical methods was not easily influenced by inliers or outliers; 2) the Russel and Rao Index produced the most constant groupings of ART clinics compared to the Jaccard and Dice coefficients. This process resulted in the generation of a new categorical variable to represent cluster membership of each case in the sample.

Finally, we descriptively explored how the clusters (or models of task-shifting/task-sharing) varied by environmental factors (e.g., country, health facility type) and programmatic characteristics (e.g., time to ART initiation, ART refill schedule). Continuous variables were summarized using means and medians while categorical variables were summarized with frequencies and percentages. No statistical testing was done to compare the clusters given the small numbers of clinics (≤10) within each cluster. Limited power meant the study could only detect extreme differences of one to two standard deviations between clinic means.

### Delphi survey

We employed the Delphi Method to make systematic use of expert opinions [37] to ensure the clusters were meaningful in the context of ART services in SSA. Experts were purposefully identified through peer-reviewed publications on task-shifting/task-sharing and by nomination by colleagues in HIV care and treatment. Experts completed two structured interviews in person or via email. In the first interview, experts reviewed the cluster analysis results and completed an eight-question semi-structured questionnaire. In the second interview, experts were presented summarized results and asked to clarify and expand on their responses to the first questionnaire. All responses were analyzed and summarized thematically.

## Results

### Number of clusters or models

The hierarchical cluster analysis dendrogram and cluster fit suggested a three-cluster solution, with each cluster representing a model of ART service delivery. The 19 clinics were divided into three groups of 4, 5, and 10 clinics (Table 1). Eight of the nine sensitivity analyses yielded the same groupings of clinics, while one algorithm involving the single method yielded six clusters (data not shown).

**Table 1:** Task-shifting and task-sharing of antiretroviral therapy services, by clusters

Cadre and Roles^a^	CLUSTER 1 Traditional Model (*N* = 5)		CLUSTER 2 Mixed Model (*N* = 10)		CLUSTER 3 Task-Shifted Model (*N* = 4)	
	*n*	%	*n*	%	*n*	%
Medical doctor						
ART initial prescription	5	100	10	100	0	0
ART monitoring & management	5	100	10	100	0	0
Clinical services	5	100	10	100	0	0
Referral services	4	87	10	100	0	0
ART adherence counselling	0	0	4	40	0	0
Clinical officer						
ART initial prescription	2	40	9	90	4	100
ART monitoring & management	2	40	10	100	4	100
Clinical services	2	40	10	100	4	100
Referral services	2	40	10	100	4	100
ART adherence counselling	0	0	7	70	0	0
Nurse/ Midwife						
Registration	3	60	2	20	2	50
ART initial prescription	0	0	4	40	3	75
ART monitoring and management	2	40	6	60	3	75
Clinical services	1	20	7	70	2	50
Referral services	0	0	7	70	3	75
ART dispensing at the HIV clinic	1	20	4	40	3	75
ART adherence counselling	2	40	8	80	2	50
Phlebotomy	0	0	5	50	1	25
Patient tracing for missed appointments & defaulters	0	0	3	30	1	25
Medical records management	1	20	2	20	1	25
Counsellor						
Triage	0	0	4	40	1	25
ART adherence counselling	2	40	7	70	2	50
Patient tracing	0	0	3	30	0	0
Pharmacist						
ART dispensing at the HIV clinic	3	60	2	20	1	25
Pharmacy technician						
ART dispensing at the HIV clinic	2	40	7	70	0	0
ART adherence counselling	0	0	3	30	0	0
Laboratory technician						
Phlebotomy	2	40	7	70	3	75
Data clerk						
Registration	2	40	1	10	1	25
Patient tracing for missed appointments & defaulters	1	20	2	20	1	25
Medical records management	3	60	2	20	4	100
Lay health worker						
Registration	1	20	4	40	0	0
Patient tracing for missed appointments & defaulters at the HIV clinic	0	0	4	40	1	25
Community health worker						
Patient tracing for missed appointments & defaulters at the HIV clinic	3	60	3	30	1	25

^a^These are actual roles performed by cadres on a typical day in the ART clinic; they may vary from expected roles noted in a country policy guideline

The staffing patterns of the three clusters were examined. Two models represented task-sharing of clinical services between doctors and COs, distinguished by whether nurses played a role in clinical care or not. The third model represented complete task-shifting of clinical services from doctors to COs and nurses. Lay health workers (LHWs) task-shared with nurses and counsellors in providing adherence support to patients in all models. In two models, LHWs also provided support essential to efficient clinic flow, such as patient registration and medical file retrieval/archival.

### Expert panel response to the three models

Eighteen of the 22 invited experts participated in the survey (82% response rate). Experts had 5 to 21 years of experience implementing ART task-shifting services, policy, or research in 21 African and 9 non-African countries. They represented a range of organizations including MOHs, national and international implementation partners, universities, and funding agencies.

Most experts (14/18, 78%) said the three service delivery models identified were consistent with their knowledge and experience of ART programmes in SSA. Four experts (4/18, 22%) said model findings were “artificial” and “context dependent” and only meaningful to the three countries where data were collected.

### Model profiles

#### Overview

All three task-shifting/task-sharing models included clinics from the three study countries and from every health facility types encompassing national referral, provincial/district, and primary health centers (PHCs)) (Table 2). These findings suggested successful application of the cluster analysis, which organized facilities around staffing of ART service delivery. Models are labeled and described in detail below.

**Table 2:** Summary of contextual factors, by clusters

CLUSTER	ART Site	Facility Level	Facility Type	# Current ART Patients	Urban or Rural	Number of providers on a typical clinic day			
						# Medical doctors	# Clinical officers	# Nurses/midwives	# Lay health workers
1 Traditional Model (*n* = 5 clinics)	TZ-5	Nat Ref	Mission	1000–2000	Urban	3	3	2	0
	TZ-6	Nat Ref	Mission	<1000	Urban	5	0	3	0
	TZ-7	District	Government	2000–4000	Urban	3	2	10	2
	UG-8	Nat Ref	Non-prof	>4000	Urban	7	0	8	4
	ZM-14	Nat Ref	non-religious Government	2000–4000	Urban	3	1	2	2
2 Mixed Model (*n* = 10 clinics)	TZ-3	Provincial	Mission	1000–2000	Urban	1	2	3	2
	TZ-4	District	Government	<1000	Rural	2	2	1	1
	UG-9	PHC	Non-prof	2000–4000	Urban	1	2	3	5
	UG-10	PHC	non-religious	2000–4000	Urban	4	1	12	0
	UG-11	PHC	Mission	2000–4000	Urban	2	5	5	4
	UG-13	PHC	Non-prof	2000–4000	Urban	1	1	3	0
	ZM-15	Provincial	non-religious	>4000	Urban	1	1	2	5
	ZM-16	District	Non-prof	>4000	Urban	1	2	3	3
	ZM-17	District	non-religious	<1000	Rural	3	5	1	2
	ZM-19	Provincial	Government Mission Government Government	>4000	Urban	1	2	5	14
3 Task-Shifted Model (*n* = 4 clinics)	TZ-1	District	Government	<1000	Rural	0	1	3	0
	TZ-2	Provincial	Government	1000–2000	Urban	0	2	9	1
	UG-12	District	Government	<1000	Rural	0	1	6	8
	ZM-18	PHC	Government	2000–4000	Urban	0	2	4	10

Key: *TZ* Tanzania, *UG* Uganda, *ZM* Zambia, *Nat Ref* National Referral, *PHC* Primary Health Center

Non-prof non-religious = Non-profit, non-religious

#### Model 1-traditional model: Task-sharing of major clinical responsibilities between doctors and clinical officers (*n* = 5)

The healthcare staffing pattern of Model 1-Traditional was characterized by task-sharing of major clinical responsibilities between doctors and COs. Doctors performed most clinical tasks, including initial ART prescription, ART monitoring and management, and clinical services (all 100%; Table 1). COs were involved in some of these tasks (40%). Nurses in Model 1-Traditional were not responsible for prescribing ART. Nurses provided clinical services (20%) and ART dispensing (20%) at a few clinics, but generally they were responsible for patient registration (60%) and ART adherence counselling (40%). LHWs also provided clinic- and community-based adherence support, and pharmacists dispensed ART instead of pharmacy technicians.

In this traditional model, 80% of clinics were located in national referral hospitals and 100% in urban centers (Table 2). They were led by mission, government, or non-profit, non-religious organizations. The number of ART patients varied from <1000 to >4000, and there were on average 4 doctors, 1 CO, 5 nurses/midwives, and 2 LHWs on a typical day (Table 2).

We identified several distinguishing features of ART service delivery in Model 1-Traditional clinics: a shorter time to ART initiation, greater access to laboratory testing services, fewer integrated supportive services for TB, and nutritional supplementation, and no community-based ART distribution.

#### Shorter time to ART initiation

All clinics in Model 1-Traditional determined ART eligibility in under 7 days whereas ≥50% of ART clinics in the other models took more than 7 days. Model 1-Traditional clinics also took less time to initiate patients on ART: only 20% took more than 7 days compared to 40–50% in the other models. Further, less than half of Model 1-Traditional clinics required patients to participate in 3 or more counselling sessions before initiating ART compared to 75–90% of clinics in the other two models (Table 3). Some physician experts hypothesized that doctors had greater clinical expertise to make decisions on ART eligibility and could initiate ART more quickly than nurses who may rely on standardized clinical decision-making tools. However, nurse experts countered this argument, noting that these traditional model clinics were relatively well-resourced tertiary-level facilities. Facilities with more resources were more likely to staff the clinics with pharmacists who could better manage drug stocks and facilitate a shorter time to ART initiation. The idea that more well-resourced clinics can be staffed with pharmacists or pharmacy technicians trained in drug stock management is supported by few reported ART stock-outs in Model 1-Traditional (20%) compared to Model 3-Task-shifted (100%).

**Table 3:** Summary of HIV care and treatment programmatic factors, by clusters

PROGRAMMATIC FACTORS	CLUSTER 1 Traditional Model (*n* = 5 clinics)	CLUSTER 2 Mixed Model (*n* = 10 clinics)	CLUSTER 3 Task-Shifted Model (*n* = 4 clinics)
Clinic days Per Week (average clinic day is 7 h)			
Average number of clinic days at the main ART site (min, max)	4.6 (3,6)	4.4 (2,6)	4.8 (4,5)
Average number of clinic days at outreach sites (min, max)	3.0 (0,5)	1.3 (0,5)	0.5 (0,2)
Total average number of clinic days (min, max)	7.6 (3,11)	5.7 (4,10)	5.3 (5,6)
Time to ART initiation			
> 7 days to determine eligibility	0/5 (0%)	6/10 (60%)	2/4 (50%)
> 7 days to initiate AT if found eligible	1/5 (20%)	4/10 (40%)	2/4 (50%)
Site requires at least 3 counselling sessions before ART initiation	2/5 (40%)	9/10 (90%)	3/4 (75%)
One or more stock out of ART in the past 6 months	1/5 (20%)	0/10 (0%)	4/4 (100%)
Time to Less Frequent ARV Drug Refill (every 2 months instead of monthly)			
In the first month on ART	0/5 (0%)	0/10 (0%)	0/4 (0%)
Between two and six months on ART	0/5 (0%)	2/10 (20%)	0/4 (0%)
After six months on ART	1/5 (20%)	8/10 (80%)	1/4 (25%)
Frequency of laboratory testing after 6 months on ART			
CD4 cell count			
Every 3 months	1/5 (20%)	2/10 (20%)	0/4 (0%)
Every 6 months	4/5 (80%)	8/10 (80%)	4/4 (100%)
Viral load testing			
Never	2/5 (40%)	6/10 (60%)	3/4 (75%)
As needed	3/5 (60%)	4/10 (40%)	1/4 (25%)
Adherence support through the HIV care and treatment clinic			
Require treatment supporter after ART initiation	5/5 (100%)	8/10 (80%)	3/4 (75%)
Pill count during 1st six months on ART	0/5 (0%)	5/10 (50%)	3/4 (75%)
Pill count after 1st six months on ART	0/5 (0%)	5/10 (50%)	2/4 (50%)
Pill count after patient misses ARV drug refill appointment	4/5 (80%)	7/10 (70%)	3/4 (75%)
People Living with HIV support group	5/5 (100%)	8/10 (80%)	4/4 (100%)
Adherence support worker	4/5 (80%)	8/10 (80%)	3/4 (75%)
Home-based care worker	3/5 (60%)	5/10 (50%)	2/4 (50%)
Community-based services linked to the HIV care and treatment clinic			
ARV drug distribution by providers or lay volunteers	0/5 (0%)	3/10 (30%)	1/4 (25%)
Adherence support	5/5 (100%)	8/10 (80%)	4/4 (100%)
Emotional or social support	4/5 (80%)	8/10 (80%)	3/4 (75%)
Follow-up of missed appointments	4/5 (80%)	7/10 (70%)	3/4 (75%)
Nutritional supplementation	3/5 (60%)	5/10 (50%)	3/4 (75%)
Home based care	4/5 (80%)	7/10 (70%)	4/4 (100%)
Referral for medical care	4/5 (80%)	7/10 (70%)	4/4 (100%)
Follow-up of missed clinic or pharmacy appointment			
Telephone contact	4/5 (80%)	9/10 (90%)	4/4 (100%)
House visit by adherence support worker	3/5 (60%)	8/10 (80%)	3/4 (75%)
House visit by healthcare worker	1/5 (20%)	5/10 (50%)	1/4 (25%)
House visit by home-based care worker	3/5 (60%)	7/10 (70%)	3/4 (75%)
Tracing of patients loss to follow-up			
Telephone contact	4/5 (80%)	9/10 (90%)	4/4 (100%)
House visit by adherence support worker	4/5 (80%)	8/10 (80%)	2/4 (50%)
House visit by healthcare worker	3/5 (60%)	5/10 (50%)	1/4 (25%)
House visit by home-based care worker	3/5 (60%)	7/10 (70%)	1/4 (25%)

#### Greater access to laboratory testing services, especially viral load testing (VLT)

All Model 1-Traditional clinics had access to on-site laboratory services for routine patient monitoring, including CD4 cell count, liver function, renal function, white blood cell count and differential, and hemoglobin/hematocrit tests (Table 3). A greater percentage of Model 1-Traditional clinics reported access to VLT for suspected treatment failure compared to the other two models (60% vs. 25–40%; Table 3). Experts suggested that clinics with doctors and COs providing major clinical care were more likely to provide VLT as most were tertiary level facilities with laboratory access and physician capacity to interpret test results.

#### Lower proportion of supportive services for TB, nutrition, and community-based ART distribution

Only 60% of Model 1-Traditional clinics offered TB testing and treatment in the ART clinics, compared to the other two models where 90–100% offered TB testing and 75–90% offered TB treatment (Table 3). Less than half (40%) of Model 1-Traditional clinics provided community-based nutritional supplementation compared to 50–75% in the other two models. While community-based ART distribution was rare across all three models, none of the Model 1-Traditional clinics used this approach (0% vs. 25–30%).

#### Model 2-mixed model: Task-sharing of major clinical responsibilities between doctors, clinical officers, and nurses (*n* = 10)

The healthcare staffing pattern in Model 2-Mixed was characterized by doctors and COs sharing nearly equal responsibilities and nurses playing a large clinical role. Doctors and COs had almost the same level of responsibilities for ART prescription (100% doctors vs. 90% COs), ART monitoring and management (100%) and clinical services (100%); they varied in provision of ART adherence counselling (40% doctors vs. 70% COs, Table 1). Nurses had a large clinical role in ART prescription (40%), ART dispensing (40%), provision of clinical services (70%) and referral services (70%). Like Model 1-Traditional, Model 2-Mixed nurses were largely responsible for adherence counselling (80%, Table 1). In contrast to Model 1-Traditional, Model 2-Mixed clinics relied on pharmacy technicians instead of pharmacists to dispense ART. Nearly half of the clinics in the Model 2-Mixed used LHWs to register patients and trace those lost to follow-up (LTFU).

Model 2-Mixed clinics represented the largest programmes with >4000 patients on ART per clinic. They were found in provincial/district level facilities or PHCs located in urban areas. Clinics were managed by missions, governments, and non-profit non-religious organizations. There were 2 doctors, 2 COs, 8 nurses/midwives, and 4 LHWs on an average day (Table 2).

The most distinguishing feature of ART service delivery in Model 2-Mixed was strategies for managing high patient volume, including task-shifting patient registration and tracing to LHWs and extending ART refill times or community-based ART distribution.

#### Task-shifting patient registration and tracing to LHWs

In contrast to Models 1-Traditional and 3-Task-shifted where LHWs primarily provided adherence support, Model 2-Mixed had the greatest proportion of sites where LHWs held additional responsibilities in both patient registration and tracing missed appointments (40% vs. 0%–25% for both).

#### Extending length of ART refill or community-based ART distribution to reduce client flow

Model 2-Mixed Clinics were more likely to reduce client flow by providing a longer ART supply (80% provided two-month drug supplies compared to 60% in Model 1-Traditional and 25% in Model 3-Task-Shifted; Table 3). Also, a larger proportion of Model 2-Mixed clinics distributed ART in the community (30% vs. 0–25%).

#### Model 3-task-shifted model: Task-shifting of major clinical responsibilities from doctors to clinical officers and nurses (*n* = 4)

The healthcare staffing pattern of Model 3-Task-Shifted was characterized by task-shifting of clinical responsibilities from doctors to COs and nurses. There were no doctors in these clinics. COs were responsible for all clinical tasks, including ART prescription (100%), ART monitoring and management (100%), clinical services (100%), and referral services (100%). Substantial task-sharing between COs and nurses occurred: nurses in three-quarters of Model 3-Task-Shifted clinics prescribed ART, provided referral services, and dispensed ART; and nurses in half of the clinics provided clinical services as well. The only clinical tasks not shared among COs and nurses were management of drug resistant infection and monitoring of viral load results (100% CO vs. 0% nurses, Table 1). Similar to Model 1-Traditional and in contrast to Model 2-Mixed, COs did not provide ART adherence counselling; nurses and counsellors provided all adherence counselling services. LHWs also conducted adherence support in clinics (75%) and communities (75%).

Model 3-Task-Shifted included clinics based at district or provincial levels and in PHC. All were government facilities. Clinic sizes ranged from <1000 to 4000 ART patients. Half were based in rural locations. On a typical day, clinics had on average 2 COs, 6 nurses/midwives, and 5 LHWs.

The distinguishing aspects of ART service delivery in Model 3-Task-Shifted included: fewer clinics with structural support for patient retention and fewer on-site HIV laboratory services. However, these clinics had the greatest proportion of community-based services.

#### Reduced structural support for retention

Seventy-five percent of Model 3-Task-Shifted clinics had an appointment system in contrast to 100% in the other two models (Table 3). None were able to provide a pharmacy report to identify patients who missed ART drug refill compared to 70–80% in the other models. This model also had the smallest proportion of sites that conducted house visits to trace patients LTFU (Table 3). Experts hypothesized that given that the Model 3-Task Shifted clinics were all government supported they had less access to financial resources and technical support for pharmacy electronic medical records than clinics run by faith-based or other non-profit organizations.

#### Greater linkage to community-based services

Model 3-Task-Shifted clinics had the greatest proportion of sites providing linkages to community-based services, such as ART dispensing (75% vs. 0–60%), nutritional supplementation (75% vs. 40–50%), and home visits to patients who missed clinic appointments (75% vs. 60–70%). Correspondingly, this model had more LHWs. According to experts, these clinics may have relied more heavily on community-based services provided by LHWs because of limited resources, and to reduce clinic congestion.

## Discussion

This is the first study to identify models of ART service delivery by examining healthcare staffing patterns across clinics in multiple SSA countries. Three models were identified, distinguished primarily by the availability of doctors, nurses’ scope of clinical responsibility and the use of LHWs. The models reflected historical scale-up of ART services in SSA where ART was initially available only at tertiary-level hospitals in urban centers (Model 1-Traditional) and was later decentralized to peripheral health facilities in peri-urban towns and rural villages, such as district-level hospitals and PHCs (models 2-Mixed and 3-Task-Shifted). In Model 1-Traditional, doctors and COs took the lead on initiating and clinically managing ART patients, and nurses had limited clinical responsibilities. None of the nurses in Model 1-Traditional prescribed ART; instead, nurses were mainly responsible for patient registration and adherence counselling. This model most closely resembled the earliest approaches to HIV service delivery prior to decentralization [10, 38–40]. Model 1-Traditional clinics received substantial financial, technical, and infrastructural support from external donors. Many of these clinics are now considered clinical centers of excellence where patients can access advanced treatment and laboratory services need to manage third line regimens and resistance testing.

In Model 2-Mixed, fewer doctors were available and nurses had more clinical responsibilities, such as prescribing ART for treatment naïve patients and clinical monitoring of stable patients [31]. Model 2-Mixed also had more trained LHWs performing tasks such as patient registration, medical chart filing, adherence counseling and tracing of patients LTFU. These clinics match closely to the descriptions of other international non-government organization-supported ART programmes from Ethiopia [2], Malawi [4, 41], Mozambique [40], Lesotho [29, 42], and Uganda [6], where financial resources may allow the hiring of doctors. By task-sharing clinical management of stable patients to nurses and COs, doctors are freed to care for very sick or complex patients who require advanced medical knowledge and skills [2–4, 28, 29, 42–44].

Model 3-Task-Shifted did not typically have doctors present. Both COs and nurses were responsible for initiating and prescribing ART. In addition to their extensive clinical responsibilities, nurses in Model 3-Task-Shifted also performed administrative and patient support work. Nurse-initiated and/or nurse-managed ART programmes have been previously described in South Africa [3, 9, 28, 38, 43, 45–47], Rwanda [14], Lesotho [29], and Malawi [41, 48]. We believe our study’s Model 3-Task-Shifted clinics most closely resembled the Lesotho, Malawi, and Rwanda examples [14, 29, 48], and could be distinguished from the early demonstration projects of nurse-managed ART in South Africa where nurses ran “down-referral” or “step-down” ART programmes to care for clinically stable patients in the community [9, 28, 38, 45–47]. In contrast, Model 3-Task-Shifted catered to all patients, including stable patients and those with more complex health needs, such as severe side effects, rare opportunistic infections, and treatment failure. However, there may be fewer distinctions between Model 3-Task-Shifted in our study and the current implementation of nurse-initiated and nurse-managed ART in South Africa because down-referral of clinically stable patients is no longer typical – nurses initiate and manage all kinds of patients as the Government has recognized the critical role of nurses in achieving the 90–90-90 goals [49].

Our findings also concurred with observations that decentralized ART programmes, such as those in Model 2-Mixed and Model 3-Task-Shifted, required fewer clinic visits from patients [46]. For example, Model 2-Mixed and Model 3-Task-Shifted clinics provided on average 2 months of ART to stable patients to allow more time between drug refills compared to Model 1-Traditional clinics, which offered 1 month of ART only to stable patients [28, 30, 46]. Our study also found that decentralized ART programmes, such as Model 2-Mixed and Model 3-Task-Shifted clinics, were more likely than non-decentralized programmes to conduct clinic-based pill counts as part of their adherence strategies. This finding contrasts with the discussion from Grimsrud et al., where patients in a decentralized ART programme were hypothesized to have fewer opportunities for individual adherence counselling and support because down referral sites had less frequent clinic visits [46]. Our findings may differ because Grimsrud et al. described a programme for clinically stable patients who, in part, qualified for the programme by previously demonstrating good adherence.

### Implications

Our findings identified three models of HIV service delivery for potential application to other high HIV prevalence settings in SSA. Classifying ART models enables further study on their context, frequency of use, costs, and outcomes. For example, future research could assess how a task-shifted model compares to a mixed model in costs and proportion of patients achieving virologic suppression. Resulting data could inform decisions by programme planners, policy-makers, and funders on models for scale-up within resource-constrained settings.

Our study also suggested that different patterns of healthcare staffing were associated with different environmental factors and programme characteristics. For example, integrated TB treatment varied across the three service delivery models. This issue is important given high rates of TB/HIV coinfection across SSA and highlights how service provision is shaped by health facility levels, rural/urban settings, available health cadres, patient populations, and donor funding. A comprehensive and nuanced understanding of models of HIV care and treatment is especially important following WHO’s test and treat recommendation and donor fatigue after the global economic crisis. Test and treat adoption will likely impact client volumes and client composition, as healthier patients access ART. Understanding the pros and cons of different models of care can help programmes determine the best staffing patterns to adapt to increasing client loads immediate ART initiation, and the selection of different adherence strategies most effective for their facility’s patients. Concurrently, national programmes will need to identify differentiated models of care for people to meet the needs of long-term ART patients requiring more complicated treatment management, such as third line regimens and treatment for severe comorbidities (e.g., hepatitis, cancer.).

### Strengths and limitations

Our sample included 19 ART clinics in three countries – a considerably larger and more diverse sample size than past research, which has generally described task-shifting/task-sharing practices of a few clinics in one country [3, 6, 17, 18, 20, 28, 30, 40]. While this evaluation offered more information than previously available, sites were not randomly selected so data may not be generalizable to ART clinics in these countries, or SSA.

Our survey also assessed a wider range of ART service delivery components. While past research mainly described task-shifting/task-sharing in terms of who prescribed ART or provided clinical monitoring, ours accounted for other major tasks within the ART clinic (e.g. patient registration, adherence counselling, patient tracing) and the health cadres providing these tasks. While we examined the presence or absence of a wide range of services at ART clinics, a limitation is that we were unable to consider other important details, such as intensity and coverage of services per patient, or service quality.

Finally, the combination of a cluster analysis with a Delphi survey was stronger than either method alone in identifying meaningful models of ART service delivery. The Delphi survey ensured the three clusters were more than a statistical artefact and held practical meaning and had content validity.

## Conclusion

Findings highlighted the complexity of factors that need to be considered to understand effective ART programmes. These models of task-shifting/task-sharing can provide a basis for additional implementation science research, including costing analysis and comparative effectiveness across models of care, to inform the scalability and sustainability of HIV care and treatment in RLS.

## Additional file

Additional file 1: “ART Task-Shifting Survey.pdf” is the file of the structured survey used to collect task-shifting and task-sharing practices of ART services. This survey was embedded in the larger healthcare manager survey and assessed staffing practices of major ART tasks, including registration, triage, ART initial prescription, ART monitoring and management, clinical services, referral services, ART dispensing at the HIV clinic, ART drug adherence counselling, phlebotomy, follow-up on missed appointments and defaulters, medical records management, and other services. (PDF 78 kb)

## Abbreviations

ART: Antiretroviral Therapy
CO: Clinical Officer
HIV: Human Immunodeficiency Virus
IRB: Institutional Review Board
LHW: Lay Health Worker
LTFU: Lost to Follow Up
MOH: Ministry of Health
PHC: Primary Health Center
RLS: Resource Limited Settings
SSA: Sub-Saharan Africa
TB: Tuberculosis
VLT: Viral Load Testing
WHO: World Health Organization
