# Supplementary material for: Identifying models of HIV care and treatment service delivery in Tanzania, Uganda, and Zambia using cluster analysis and Delphi survey
Source: BMC Health Serv Res. 2017 Dec 6;17:811. doi: 10.1186/s12913-017-2772-4 (PMC5717830; doi:10.1186/s12913-017-2772-4)
Supplement: Additional file 1: — “ART Task-Shifting Survey.pdf” is the file of the structured survey used to collect task-shifting and task-sharing practices of ART services. This survey was embedded in the larger healthcare manager survey and assessed staffing practices of major ART tasks, including registration, triage, ART initial prescription, ART monitoring and management, clinical services, referral services, ART dispensing at the HIV clinic, ART drug adherence counselling, phlebotomy, follow-up on missed appointments and defaulters, medical records management, and other services. (PDF 78 kb) [file 12913_2017_2772_MOESM1_ESM.pdf]

14.	*Read aloud:* For the following questions, we are interested learning about the staffing of current services and whether there have been any changes in type of staffing since the start of this ART programme. Changes in staffing may take place formally with directions from the Ministry of Health or NGO/ clinic management, or it may take place informally based on the availability of personnel with skills and time at the clinic. Examples of a change in type of staffing include: doctor to nurse prescription of ART, or nurse to lay support worker for ART adherence.									
		I	II	III	IV	V	VI	VII	VIII	
	HIV Clinic Service	How many staff currently do <insert service> on a typical day at the HIV clinic?	Specify type of staff* are currently providing this service	Has there ever been a change in the type of staff providing this service, including lay workers?	If yes, specify type of staff* who has previously provided this service	What month and year did this change take place	Is there a document / standard operating procedure for this staff change?	Were staff trained before starting the new task?	Have staff received refresher training/ mentorship since starting the new task?	*Staff Type Categories (for columns II and IV)
a.	Registration			00 No<br>01 Yes		___/___<br>(M/Y)	00 No<br>01 Yes	00 No<br>01 Yes	00 No<br>01 Yes	01 Medical doctor/ officer<br>02 Clinical officer/ assistant<br>03 Nurse/ midwife (enrolled/ registered)<br>04 Lay ART support worker (non-medically trained, based primarily at the clinic, but not on the formal payroll)<br>05 Nutritionist<br>06 Social workers<br>07 Pharmacist<br>08 Pharmacy technician/ assistant<br>09 ART counsellor<br>10 Lab technician/ assistant<br>11 Data entry clerk<br>66 Other (specify)
b.	Triage (patient screening at entry to determine why the patient has come to the ART clinic and what services are needed to meet patient needs, i.e. critical symptoms requiring emergency care, routine care)			00 No<br>01 Yes		___/___<br>(M/Y)	00 No<br>01 Yes	00 No<br>01 Yes	00 No<br>01 Yes	
c.	ART initial prescription (starting a patient on ART based upon assessment for ART eligibility)			00 No<br>01 Yes		___/___<br>(M/Y)	00 No<br>01 Yes	00 No<br>01 Yes	00 No<br>01 Yes	

14.		I	II	III	IV	V	VI	VII	VIII	
	HIV Clinic Service	How many staff currently do <insert service> on a typical day at the HIV clinic?	Specify type of staff* are currently providing this service	Has there ever been a change in the type of staff providing this service, including lay workers?	If yes, specify type of staff* who has previously provided this service	What month and year did this change take place	Is there a document / standard operating procedure for this staff change?	Were staff trained before starting the new task?	Have staff received refresher training/ mentorship since starting the new task?	*Staff Type Categories (for columns II and IV)
d.	ART monitoring and management (ongoing assessment of patient's response to ART, including diagnosis and management of any drug side effects; clinical (emergence of new OIs), immunological (CD4 count), and if available, virological (viral load count) monitoring to determine if ARV regimen is efficacious; and diagnosis and management of drug resistance (switch to 2nd line regimen)			00 No<br>01 Yes		___/___<br>(M/Y)	00 No<br>01 Yes	00 No<br>01 Yes	00 No<br>01 Yes	01 Medical doctor/ officer<br>02 Clinical officer/ assistant<br>03 Nurse/ midwife (enrolled/ registered)<br>04 Lay ART support worker (non-medically trained, based primarily at the clinic, but not on the formal payroll)<br>05 Nutritionist<br>06 Social workers<br>07 Pharmacist<br>08 Pharmacy technician/ assistant<br>09 ART counsellor<br>10 Lab technician/ assistant<br>11 Data entry clerk<br>66 Other (specify)
e.	Clinical services (diagnosis and management of OIs, provision of Cotrimoxazole prophylaxis, and management of non-HIV conditions, i.e. malaria, broken bones)			00 No<br>01 Yes		___/___<br>(M/Y)	00 No<br>01 Yes	00 No<br>01 Yes	00 No<br>01 Yes	

14.		I	II	III	IV	V	VI	VII	VIII	
	HIV Clinic Service	How many staff currently do <insert service> on a typical day at the HIV clinic?	Specify type of staff* are currently providing this service	Has there ever been a change in the type of staff providing this service, including lay workers?	If yes, specify type of staff* who has previously provided this service	What month and year did this change take place	Is there a document / standard operating procedure for this staff change?	Were staff trained before starting the new task?	Have staff received refresher training/ mentorship since starting the new task?	*Staff Type Categories (for columns II and IV)
f.	Referral services			00 No<br>01 Yes		___/___<br>(M/Y)	00 No<br>01 Yes	00 No<br>01 Yes	00 No<br>01 Yes	01 Medical doctor/ officer<br>02 Clinical officer/ assistant<br>03 Nurse/ midwife (enrolled/ registered)<br>04 Lay ART support worker (non-medically trained, based primarily at the clinic, but not on the formal payroll)<br>05 Nutritionist<br>06 Social workers<br>07 Pharmacist<br>08 Pharmacy technician/ assistant<br>09 ART counsellor<br>10 Lab technician/ assistant<br>11 Data entry clerk<br>66 Other (specify)
g.	ART dispensing at the HIV clinic			00 No<br>01 Yes		___/___<br>(M/Y)	00 No<br>01 Yes	00 No<br>01 Yes	00 No<br>01 Yes	
h.	ARV drug adherence counselling			00 No<br>01 Yes		___/___<br>(M/Y)	00 No<br>01 Yes	00 No<br>01 Yes	00 No<br>01 Yes	
i.	Phlebotomy			00 No<br>01 Yes		___/___<br>(M/Y)	00 No<br>01 Yes	00 No<br>01 Yes	00 No<br>01 Yes	
j.	Follow-up on missed appointments and defaulters			00 No<br>01 Yes		___/___<br>(M/Y)	00 No<br>01 Yes	00 No<br>01 Yes	00 No<br>01 Yes	
k.	Medical records management			00 No<br>01 Yes		___/___<br>(M/Y)	00 No<br>01 Yes	00 No<br>01 Yes	00 No<br>01 Yes	
l.	Any other? (Specify)			00 No<br>01 Yes		___/___<br>(M/Y)	00 No<br>01 Yes	00 No<br>01 Yes	00 No<br>01 Yes	
